# Effects of Melatonin Administration on Chemical Pregnancy Rates of Polycystic Ovary Syndrome Patients Undergoing Intrauterine Insemination: A Randomized Clinical Trial

**DOI:** 10.22074/ijfs.2019.5717

**Published:** 2019-07-14

**Authors:** Fataneh Mokhtari, Firouzeh Akbari Asbagh, Ozra Azmoodeh, Mahmood Bakhtiyari, Amir Almasi-Hashiani

**Affiliations:** 1 Department of Obstetrics and Gynecology, IVF Unit, Yas Hospital, Tehran University of Medical Sciences, Tehran, Iran; 2 Non-communicable Diseases Research Center, Alborz University of Medical Sciences, Karaj, Iran; 3 Department of Community Medicine, School of Medicine, Alborz University of Medical Sciences, Karaj, Iran; 4 Department of Epidemiology and Reproductive Health, Reproductive Epidemiology Research Center, Royan Institute for Reproduc- tive Biomedicine, ACECR, Tehran, Iran

**Keywords:** Mature Follicle, Melatonin, Polycystic Ovary Syndrome, Pregnancy Rate

## Abstract

**Background:**

Oxidative stress as a potential cause of poor oocyte quality can influence a female’s reproductive system. This study aimed to investigate the effects of melatonin on chemical pregnancy rates of a significant number of polycystic ovary syndrome (PCOS) patients undergoing intrauterine insemination (IUI).

**Materials and Methods:**

In this double-blinded randomized clinical trial (RCT) study, the samples included 198 PCOS patients fulfilling the inclusion criteria and undergoing the IUI treatment. On the third day of menstruation, a 3-mg melatonin tablet or its placebo was given to the patients according to the randomized study protocol; this prescription was continued until the day of human chorionic gonadotropin (hCG) administration. The current study attempted primarily to scrutinize the effect of melatonin administration on the rate of chemical pregnancy and mature follicles during the IUI treatment cycle, and secondarily to determine the endometrial thickness (ET) on the day of IUI.

**Results:**

The mean age of the participants in the study was 28.9 ± 5.5 years. The chemical pregnancy rate in the group receiving melatonin was about 32%, when it was 18% in the control group (P=0.012). Furthermore, it was concluded that the addition of melatonin to the treatment cycle of PCOS individuals could significantly improve the ET after the treatment (P<0.001).

**Conclusion:**

The results of this study demonstrated that the treatment of PCOS patients undergoing IUI with mela- tonin significantly improves the rate of chemical pregnancy (Registration number: IRCT2017021132489N1).

## Introduction

As one of the most common endocrine disorders, polycystic ovary syndrome (PCOS), has complex pathophysiological characteristics, which have not yet been understood completely. PCOS involves about 5-10% of women of reproductive age and it seems to be an important cause of infertility (1). It is worth mentioning that the quality of oocytes plays a pivotal role in the development of clinical pregnancies. It has been reported that in humans a cause of infertility in women and an essential obstacle to successful in vitro fertilization (IVF) is the poor quality of oocytes (2). In many inclusive investigations, numerous therapeutic strategies have been suggested for patients with repeated implantation failures, such as hysteroscopy, endometrial injury, stimulation protocol modification, blastocyst transfer, assisted hatching, pre-implantation genetic screening for aneuploidy, and the supplementation of vitamins and antioxidants (3-7). According to the present reports, the probability of achieving a live birth after an assisted reproductive technology (ART) cycle is approximately 30% (8). Moreover, it should be mentioned that quite a few strategies have been examined over time to improve this rate (6, 9, 10).

Oxidative stress as a potential cause of poor oocyte quality can influence female reproduction (11). Current investigations have discovered that melatonin acts as a free radical scavenger and stimulates antioxidant enzymes, so it protects cells from oxidative stress (12, 13). Therefore, melatonin supplementation can protect oocytes from oxidative stress leading to the unsuccessful reproductive outcomes of women undergoing ART (14). A number of clinical trials have depicted that melatonin supplementation with or without other treatments has been considered as a valuable approach to improve the quality of oocytes and the outcomes of IVF in both PCOS patients and normal women (15-17).

Consequently, the aim of the present study was to investigate the effect of melatonin on the rate of chemical pregnancy among PCOS patients undergoing intrauterine insemination (IUI).

## Materials and Methods

### Study design and sample size

**Fig 1:**
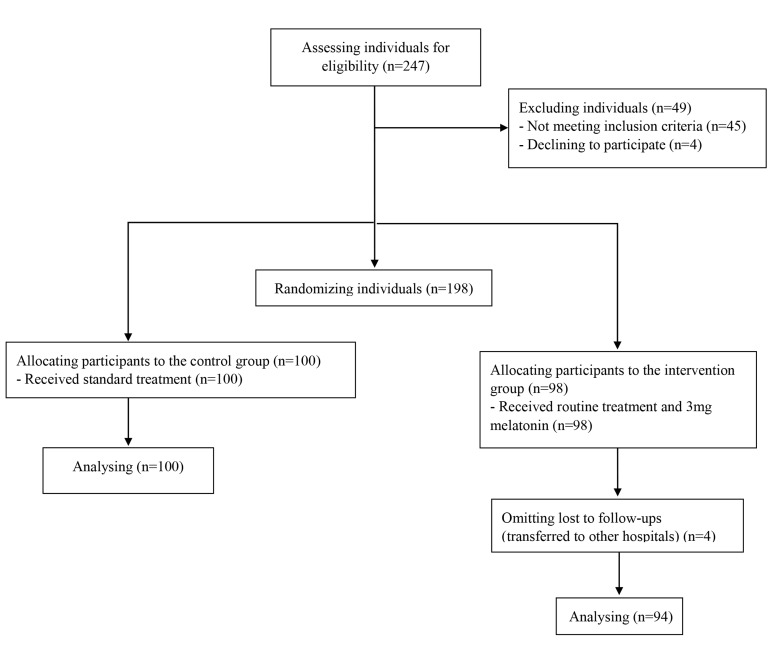
Flowchart of the study

This study was performed as a randomized, double-blinded clinical trial study (RCT) with a parallel-groups design. It was carried out using a 1:1 allocation ratio for the intervention group receiving melatonin and the controls receiving placebo at Yas Hospital in Tehran, Iran. The study population consisted of the PCOS-diagnosed patients who had been referred to the hospital due to infertility problems from March 2017 to September 2017. The sample of this study contained 198 patients with PCOS, meeting our inclusion criteria to participate in the study, and being recommended to undergo an IUI treatment by their physicians (Fig .1). The written informed consent was obtained from all individual participants included in the study. The proposal of this research has been approved in the Ethics Committee of Tehran University of Medical Sciences (Ethics committee code: 25667).

### Inclusion and exclusion criteria

The inclusion criteria were the following: i. Being aged between 20 to 40 years, ii. Having husbands with normal spermograms, iii. Having normal hysterosalpingography, iv. Having the Rotterdam diagnostic criteria for PCOS, v. Having no underlying endocrine diseases, and vi. Using no hormonal drugs within the past three months. Furthermore, the exclusion criteria included the following: i. Being deficient in an adequate ovarian response, ii. Suffering from ovarian hyperstimulation syndrome, and iii. No history of treatment for infertility.

### Random allocation

In the present study, a random allocation was performed, and the participants were divided into an intervention group receiving melatonin and a control group receiving placebo using a balanced block randomization technique. Considering blocks of 4 in this study, the Stata software was used to generate random-number sequences from 1 to 6 until the desired sample size was achieved. Since the total number of modes to set two people in the blocks of 4 was 6 modes, if the generated number exceeded 6, the next number was regenerated regardless of the previous number. Preparing the random allocation sequences of the participants, putting them in sealed airtight envelopes, and numbering them with a five-digit serial number were all performed by a third person who was not involved in the study design. All the envelopes (n=188) having a random 5-digit serial number were opened immediately after the completion of basic information and examination of the participants. Then, the participants were assigned to the intervention or control groups.

### Primary and secondary outcomes measured

In this study, the primary outcome was the determination of the rate of chemical pregnancies during the IUI treatment cycle and the secondary outcome was the determination of endometrial thickness (ET) on the day of IUI.

### Sample size

Considering a type 1 error of 5%, a study power of 80%, and a difference of 20% between the chemical rates in the intervention and control groups, the sample size was estimated to be 93 patients in each group.

### Treatment procedure

After initial evaluations by the physician, a basal vaginal ultrasound of the uterus and ovaries was performed during the days 1 to 3 of the menstrual cycle, using a Siemens ultrasound equipment (ACUSON X600, Germany), and the numbers of both antral follicles (AFC) and ET were recorded. Then, to induce ovulation two clomiphene citrate 50-mg tablets (Iran Hormone Company, Iran) were administered from cycle days 3 to 7. Furthermore, on the 3rd day of menstruation, a 3-mg melatonin tablet (Nature Made, USA) or its placebo (made by Faculty of Pharmacy, Tehran University of Medical Sciences) was given to the patients according to the randomized study protocol, and this prescription was continued until the day of hCG administration On the 10th day of menstrual cycle, another ultrasound was performed for both groups, and the thickness of the endometrium, AFC, and follicle sizes were recorded in millimeters. Subsequently, based on the information obtained at this stage, the need for 75 IU of human menopausal gonadotropin (HMG 75, Menogon, Germany) injection was estimated and it was injected intramuscularly by a trained nurse.

Vaginal ultrasound was performed periodically according to the needs of each patient by the physician until a suitable follicle size (i.e., greater than or equal to 18 mm) was seen. If the appropriate size of the follicle was observed, at the same time, estradiol was measured using Monobine kits and two hCG 500 ampoules (Choriomon, Swiss) were injected intramuscularly.

After the above steps, which took about 36-40 hours, the patients referred to the clinic and underwent IUI. Six days later, blood progesterone levels were measured by DRG Progesterone ELISA Kit (Marburg, Germany). Then, 14 days after IUI, hCG-B blood test (GenWay Biotech. Inc, USA) was performed using an antibody kit to determine the rate of chemical pregnancy. 

### Statistical analysis

The normality assumption was assessed by the Kolmogorov- Smirnov test. Mean ± standard deviation (SD) and median (inter-quintile range) were used for presenting data with normal and non-normal distributions, respectively. Differences between the two groups of participants were assessed using independent Student’s t tests. We used the Mann-Whitney test for continuous variables and chi-square tests for categorical variables. To evaluate the differences between the means of endometrial diameters in the intervention and control groups, an analysis of covariance with the adjustment of baseline scores was used. The Stata software (StataCorp LLC, version 13MP) was utilized to perform all the statistical analyses. Data with P<0.05 were considered statistically significant.

### Results

In the present study, the information on the clinical infertility treatments of 198 infertile patients referring to Yas Hospital in Tehran, Iran, were used (Fig .1). The mean age of the participants in the study was 28.9 ± 5.5 years. In this study, 94 patients received melatonin as the intervention group and 100 patients received placebo as the control group. The results of comparing basic and clinical features of the participants in the study revealed that these two groups did not show any significant differences in the basic features at the time of entering the study. Table 1 summarizes their basic and clinical information.

**Table 1:** Basic and clinical features of the patients in the intervention and control groups


Variables	Intervention group (n=94)	Control group (n=100)	P value

Age (Y)	28.4 ± 5.5	29.3 ± 5.6	0.241
BMI (Kg/m^2^)	27.6 ± 4.0	28.1 ± 3.7	0.056
Infertility duration (Month)	35.1 ± 21.7	43.5 ± 25.7	0.015
Primary endometrial thickness (mm)	4.6 ± 0.56	4.4 ± 0.52	0.093
Estradiol concentration (pg/ml)	1730 ± 281	1850 ± 534	0.001
IUI cycle duration (Day)	17.2 ± 2.6	16.8 ± 2.1	0.371
Total follicle count (n)	24.6 ± 4.7	23.9 ± 4.6	0.323
Infertility type			0.581
Primary infertility (%)	86 (91.5)	94 (94)	
Secondary infertility (%)	8 (8.5)	6 (6)	


Data are presented as mean ± SD or n (%)

Regarding the evaluation of the serum concentration of estradiol in both intervention and control groups, the results represented the mean of this hormone concentration in the control group as 1850 ± 534 pg/ml and in the intervention group as 1730 ± 281 pg/ml. An independent t test indicated that the two groups had significantly different levels of serum estradiol concentration (P=0.001).

The IUI cycle in the intervention group lasted for 17.2 ± 2.6 days and in the control group lasted for 16.8 ± 2.1 days. The result showed that there was no significant difference between the two groups (P=0.371). Furthermore, the results of Mann-Whitney test confirmed that there were no statistically significant differences between the rates of HMG doses used within the two groups (P=0.970). 

In Table 2, the results of the ET, the number of mature follicles, and the chemical and clinical pregnancy rates are compared between the two groups. As demonstrated in Table 2, the chemical pregnancy rate in the group receiving melatonin was about 30%, while this value was 18% in the control group. The chi-square test indicated that the difference between these two values was statistically significant (P=0.011). Also, the addition of melatonin to the treatment cycles of PCOS individuals significantly improved the sizes of follicles during the IUI cycles (P=0.002). Regarding the mean ET of the patients, our covariance analysis showed that there was a significant difference between the intervention and control groups (P<0.001). The mean ET of the patients in the intervention group increased more than that in the controls. 

**Table 2:** Comparison of IUI outcomes between the intervention and control groups


Variables	Intervention group (n=94)	Control group (n=100)	P value

Melatonin concentration (pg/ml)	190.7 ± 34.1	74.5 ± 17.1	<0.001^a^
Mature follicle (n)	2 (2-3)^b^	2 (1-3)	0.002^c^
Endometrial thickness after the treatment (mm)	9.2 ± 1.33	8.5 ± 0.87	<0.001^d^
Chemical pregnancy (%)	30 (32)	18 (18)	0.012^e^
Clinical pregnancy (%)	26 (27.6)	15 (15)	0.013^e^


Data are presented as mean ± SD or n (%). IUI; Intrauterine insemination, ^a^; Independent sample t test, ^b^; Median and IQR, ^c^; Mann-Whitney test, ^d^; ANCOVA test with adjusting baseline Endometrial thickness, and ^e^; Chi-square test.

## Discussion

The results of the present study suggest that following IUI, melatonin treatment has a favorable effect on mature follicles, ET, as well as chemical and clinical pregnancies in infertile PCOS women. 

In recent studies, it has been shown that oxidative stress has an adverse effect on infertility treatments, so researchers are trying to find possible mechanisms of preventing these unfavorable effects (18). In this case, melatonin, an indoleamine synthesized from tryptophan, is a new oxygen scavenger, which can be used to improve pregnancy outcomes in infertile women (19, 20).

Studies at the molecular level have discovered that in pregnant rats, melatonin supplementation improves serum 17ß-estradiol levels. Also, in rat uterine tissue, it enhances the expression of MT(1), MT(2) melatonin receptors, p53 receptor, and consequently may improve the uterine environment, thus playing an important role in embryo implantation at least in rats (21). However, it should be mentioned that based on Succu et al. (22), a high concentration of melatonin in embryo culture media could be harmful, as it displays a degree of toxic activity on embryos.

In the present study, the results suggested that adding melatonin to the IUI treatment cycle could significantly improve the quality of follicles during the cycle in PCOS cases. Along with our findings, Eryilmaz et al. (23) in an un-blinded randomized controlled trial on IVF patients who were also suffering from sleeping disorders, observed a significant increase in the number of retrieved oocytes, metaphase II (MII) oocyte and grade 1 embryos after the prescription of 3-mg melatonin from days 3-5 until HCG injection. Batioglu et al. (24) revealed a higher percentage of MII oocytes and grade 1 embryos in a melatonin- treatment group. However, in their study, no significant difference was reported in the number of oocytes in women who underwent IVF cycles. Also a large number of studies have concluded that melatonin has a useful effect on retrieved oocytes, MII oocytes, and good quality embryos (17, 25, 26).

In a recent study, Jahromi et al. (27) showed that in women with diminished ovarian reserve, the mean of grade 1 embryos and mature MII oocytes were significantly higher in the melatonin-treatment group in comparison with the control group, but there was no significant difference in other ART outcomes, such as grade 2 embryos and metaphase I (MI) oocytes. It was also reported that oxidative stress had an adverse effect on oocyte maturation and melatonin supplementation, protecting oocytes against oxidative stress (28). Nikmard et al. (29), in a study on mouse models, concluded that melatonin could significantly improve nuclear maturation of PCOS oocytes.

The results of the current study also revealed that the mean of ET in the melatonin-treatment group increased more than that in the control group. Therefore, melatonin has a favorable effect on ET in infertile PCOS patients following IUI. Unfortunately, there are not many studies on the relationship between melatonin and ET, but based on an animal study on rats, it is suggested that melatonin can affect the endometrial morphology and increase embryo implantation (30).

In the present study, chemical and clinical pregnancy rates were significantly higher in the melatonin-treated group compared to the placebo group. In a systematic review and meta-analysis of five published randomized controlled trials conducted by Seko et al. (31), a pooled risk ratio of 1.21 for the clinical pregnancy rate in favor of melatonin was revealed and this pooled risk ratio turned out to be significant [95% confidence interval (CI) 0.98-1.50]. 

In a relatively large randomized controlled trial in PCOS infertile cases undergoing intracytoplasmic sperm injection, researchers compared outcomes in two groups including myo-inositol 4g, folic acid 400mcg and melatonin 3mg per day (n=178) and myo-inositol and folic acid alone (n=180). The results showed that patients in the first group had greater numbers of mature oocytes and grade 1 embryos. These results, therefore, support the positive effect of melatonin in the treatment of PCOS infertile women (32).

In the present study, which was an attempt to provide novel information on the effects of melatonin on PCOS patients of a certain age group, some limitations existed, including the difficulty of frequent ultrasound scanning for patients until the right size of follicle was seen, and also patients’ poor cooperation with the treatment due to their unfamiliarity with melatonin prescription. Besides, the study was conducted on PCOS cases with the mean age of 28.9 years old and just IUI cycles were included in this study. Therefore, the obtained results are generalizable to infertile women suffering from PCOS in a relatively young age group. It is suggested to conduct a multi-center RCT to obtain more generalizable and valid results. 

## Conclusion

The results of this study demonstrated that the treatment of PCOS patients undergoing IUI with melatonin can significantly improve the quality of follicles and, as a consequence, the rate of chemical pregnancies.
